# Populatory Calculations

**Published:** 1859-08

**Authors:** 


### POPULATORY CALCULATIONS.

If the United States maintains the rate of population of the last fifteen years, the number of inhabitants for the year of grace nineteen hundred, will amount to one hundred and ten millions. If our country was as thickly settled as Russia, it would have . . . . . . . . . . . . . . 80,000,000
As New England, . . . . . . . . . . . 123,000,000
Middle States, . . . . . . . . . . . . 170,000,000
France, . . . . . . . . . . . . . . 500,000,000
Great Britain, . . . . . . . . . . . . 660,000,000
Belgium, . . . . . . . . . . . . . . 1,150,000,000

But there are only a thousand million of people in the world now, hence there is room enough in the United States for all mankind; and then it would not be as thickly settled as some countries in the old world.

Reasoning from probabilities, it is not likely that any such rate of population will ever obtain on the surface of the earth, as all created material things, have within them, the elements of decay, to say nothing of war, famine, pestilence, and plagues.

But there is a general calculation of the universal prevalence of the christian religion. The natural result of a true christianity, is thrift; and the physical result of thrift, is a decrease of population. So that we need not take the trouble, nor subject ourselves to that most grievous of all self denials, of shakerism, that is, to abstain from marriage. The slaves of our own country, (like the Israelites in bondage) are more prolific than their "well-to-do" masters. Hard work promotes population; besides, the offspring of the laboring classes are more likely to live, than of those who do not labor much. Few people, comparatively speaking, who work but moderately, raise more than three or four children. In short, the statistics of all countries, and of all times, show this broad fact, that personal ease does not promote population, as much as severe labor.

One of the richest of Russian nobles was sent to Siberia many years ago; his wife was allowed the great favor of accompanying him; they were childless, and had been married for years. Hard as their taskmasters were in that dreary waste of ice and snow, the wife soon became a mother, and finally had six healthy children. This is an isolated fact, illustrating a great principle, that the less people have to work, the fewer children they will have; and as christianity promotes thrift, and moderations of views, the effect will be to counteract any over-peopling; in other words, the Almighty has constituted such a system of checks and balances in the government of the physical and moral universe, that there never can be a collision of interests, but universal harmony will prevail for unending ages.

